# Longitudinal Cognitive Changes in Cerebral Small Vessel Disease: The Effect of White Matter Hyperintensity Regression and Progression

**DOI:** 10.1212/WNL.0000000000213323

**Published:** 2025-02-03

**Authors:** Angela C.C. Jochems, Susana Muñoz Maniega, Una Clancy, Carmen Arteaga-Reyes, Daniela Jaime Garcia, Francesca M. Chappell, Olivia K.L. Hamilton, Ellen V. Backhouse, Gayle Barclay, Charlotte Jardine, Donna McIntyre, Iona Hamilton, Eleni Sakka, Maria Del C. Valdés Hernández, Stewart Wiseman, Mark E. Bastin, Michael S. Stringer, Michael Thrippleton, Fergus Doubal, Joanna M. Wardlaw

**Affiliations:** 1 Centre for Clinical Brain Sciences, University of Edinburgh, United Kingdom;; 2 UK Dementia Research Institute, University of Edinburgh, United Kingdom;; 3 MRC/CSO Social and Public Health Sciences Unit, School of Health and Wellbeing, University of Glasgow, United Kingdom; and; 4 Edinburgh Imaging Facility, Royal Infirmary of Edinburgh, United Kingdom.

## Abstract

**Background and Objectives:**

White matter hyperintensities (WMHs) are the commonest imaging marker of cerebral small vessel disease (SVD) and a major cause of cognitive decline and vascular dementia. WMHs typically accumulate over time, but recent studies show they can also regress, but potential clinical benefits have received little attention. We examined progressing, stable, and regressing WMH in people with stroke-related SVD and the effect on cognitive outcomes.

**Methods:**

We recruited patients with minor nondisabling ischemic stroke (modified Rankin score ≤2) from stroke services into our prospective longitudinal observational study. Participants underwent cognitive assessment and brain MRI within 3-month poststroke and 1 year later. We gathered information on vascular risk factors, stroke severity, global cognition (Montreal Cognitive Assessment [MoCA]), processing speed and executive functioning (Trail Making Test [TMT] A and B, and the B/A ratio with ratio ≥3 reflecting executive dysfunction), and the Letter Digit Substitution Test. We measured WMH volumes at baseline and 1 year and categorized net WMH volume change into quintiles: Q1 (most regression), Q3 (stable), and Q5 (most progression). We applied repeated-measures linear mixed models to analyze longitudinal WMH and cognitive changes, adjusting for age, sex, premorbid intelligence, stroke severity, disability, white matter structural integrity, and baseline WMH volume.

**Results:**

One hundred ninety-eight of 229 participants had WMH volumes available at both time-points. At baseline, the mean age was 67.5 years (SD = 10.9), with 33% female. Mean net WMH volume change per quintile was Q1 −1.79 mL (SD = 1.54), Q2 −0.27 mL (0.20), Q3 0.35 mL (0.18), Q4 1.43 mL (0.48), and Q5 5.31 mL (3.07). MoCA deteriorated the most in participants with most WMH progression (Q5) (estimated β −0.428 [95% CI −0.750 to −0.106]), compared with stable WMH (Q3), with no clear deterioration in those with most WMH regression (Q1). TMT B/A ratio improved in participants with most WMH regression (Q1; −0.385 [−0.758 to −0.012]).

**Discussion:**

WMH regression was associated with preserved global cognition and improved executive function, compared with stable WMH, while WMH progression was associated with global cognitive decline. Cognitive benefits of WMH regression suggest that WMH-affected tissue can recover, may explain variance in cognitive outcomes, offer an important intervention target, and should be assessed in other populations and longer follow-up times.

## Introduction

Cognitive decline has been related to white matter hyperintensities (WMHs) of presumed vascular origin,^1^ a common feature of cerebral small vessel disease (SVD). WMHs are associated with a range of poor clinical outcomes including an increased risk of stroke, dementia, and death.^2^ WMH-related cognitive decline can affect all cognitive domains, although is perhaps most pronounced in processing speed, attention, and executive functioning.^1^

Although generally WMHs progress over time, a recent systematic review and meta-analysis (N = 12,284) showed that WMHs also regress to some degree in up to 30% of participants.^3^ However, only 6 studies have assessed any correlates of WMH net regression, mostly focusing on imaging, hence little is known about clinical or cognitive consequences. WMH regression has been assessed in people with normal cognition and mild cognitive impairment,^4^ patients with ischemic stroke and SVD.^5-9^ Study sizes ranged from 42^8^ up to 351^4^ (total n = 1,282; 2 articles^8,9^ used the same cohort) with follow-up times of 1 year^7^ to 8.7 years.^9^

These observational studies suggest that WMH regression may associate with lower baseline WMH volumes and better microstructural integrity^10^ in total white matter,^8^ regular use of antihypertensive medication,^6^ and, at long-term follow-up, larger blood pressure reductions,^7^ less brain atrophy,^4^ a reduction in MRI measures of interstitial fluid in normal appearing white matter,^7^ and a potential improvement of memory but not of executive functioning.^4^ In 2 studies, cognitive function was comparable with that in the stable WMH group.^4,9^ One study did not find any factors related to WMH regression.^5^ However, variation in study methods limits between-study comparisons.

We aimed to examine differences in cognitive outcomes across the range of WMH net increases and decreases in patients presenting with a minor stroke, enriched for SVD features. We compared baseline clinical, cognitive, and quantitative imaging characteristics, assessed longitudinal cognitive changes, and tested the robustness of the findings to different definitions of WMH change in sensitivity analyses.

## Methods

### Participants

We recruited participants from the Lothian Stroke Services in a prospective observational cohort study.^11^ We recruited participants with a lacunar or minor (nondisabling) cortical ischemic stroke. Participants with cortical ischemic stroke act as “controls” for the lacunar-SVD stroke population since they are of similar age, have similar vascular risk factors, receive the same investigations and secondary prevention medications as those with lacunar ischemic stroke. All clinical assessments including stroke diagnosis were undertaken by specialist stroke physicians and neuroradiologists. Minor stroke was defined by a NIH Stroke Scale (NIHSS)^12^ <8 at stroke presentation, and nondisabling was defined by a modified Rankin score (mRS)^13^ of ≤2 at recruitment. Exclusion criteria were MRI contraindications, severe neurologic, cardiac, or respiratory diseases.^11^ The final stroke subtype diagnosis was based on clinical symptoms and signs, radiologic appearance on diagnostic CT and/or MRI, accounting for all available information from presentation to stroke services until study assessment. We performed detailed clinical, cognitive, and MRI study assessments within 3 months after stroke (baseline) and again 1 year later.

### Standard Protocol Approvals, Registrations, and Patient Consents

The study was approved by the Southeast Scotland Regional Ethics Committee (ref. 18/SS/0044) and conducted according to the principles expressed in the Declaration of Helsinki. All participants gave written informed consent. The study is registered under ISRCTN 12113543.

### Clinical and Cognitive Assessments

We assessed vascular risk factors including hypertension, hypercholesterolemia, diabetes mellitus, and history of smoking. Stroke severity was assessed with the NIHSS (range scores 0–42; higher scores indicate greater severity),^12^ dependency with the mRS (range scores 0 [no symptoms]–6 [death]).^13^

The cognitive assessment included the Montreal Cognitive Assessment (MoCA)^14^ for global cognition (scores range 0–30; higher scores reflect better cognition), the National Adult Reading Test (NART; baseline only) to estimate peak adult cognitive ability and estimate premorbid intelligence,^15^ and the Trail Making Test (TMT) parts A and B (time taken to complete task; faster time reflects better performance) for processing speed (part A)^16^ and executive functioning (part B).^17^ We calculated the TMT B/A ratio to assess executive functioning independent of processing speed and motor control. A B/A ratio of >3 indicates possible executive dysfunction.^17^ We additionally assessed the Letter Digit Substitution Test (LDST), a measure of information processing speed.^18^ Higher scores reflect faster processing speed.

### Imaging Acquisition

Full details of the imaging acquisition are in the published protocol.^11^ At both visits, participants underwent brain MRI on the same 3T scanner (Siemens Prisma; Siemens Healthcare, Erlangen, Germany). The images were acquired using a 32-channel head coil (Siemens Healthcare). Sequences included 3D T1-weighted (1 mm^3^ isotropic), T2-weighted (0.9 mm^3^ isotropic), fluid-attenuated inversion recovery (FLAIR) (1 mm^3^ isotropic), multishell diffusion imaging (diffusion-weighted images with b = 2,000 [64 orientations], 1,000 [64], 500 [6], 200 [3], and 0 [15] s/mm^2^; 2 mm^3^ isotropic voxels), and 3D proton density imaging (1 mm^3^ isotropic resolution). The acquisitions were repeated at the 1-year visit, but with a single-shell diffusion imaging acquisition (b = 1,000 [64], 0 [8] s/mm^2^; 2 mm^3^ isotropic voxels).

### Imaging Analysis

We coregistered all structural images to the baseline T2-weighted sequence with FLIRT^19^ from FSL.^20^ All measures were blinded to clinical and cognitive data. The intracranial volumes (ICVs) were generated computationally, checked, and manually edited if necessary. WMH volumes, defined according to the STandards for ReportIng Vascular changes on nEuroimaging criteria,^21^ were extracted from FLAIR images at both time-points using a standardized and fully developed pipeline.^7,11,22,23^ False positives and negatives were edited using Freesurfer,^24^ blinded for WMH masks at the other visit. An experienced image analyst manually segmented the index, old and acute infarcts to exclude these from the WMH volumes to avoid confounding.

We processed diffusion data with TractoR version 3.3.5 “dpreproc” pipeline.^25^ We corrected the data for susceptibility and eddy current-induced distortions. At baseline, we used the multishell diffusion-weighted volume equivalences of the 1-year single-shell acquisition. We fitted the self-diffusion tensor model using an interactive weighted least-squares approach.

We calculated peak width of skeletonized mean diffusivity (PSMD) at both visits with the publicly available pipeline for fully processed and fitted diffusion tensor images.^26,27^ We excluded index and old cortical and subcortical infarcts to avoid effects of these lesions.

### WMH Volume Change Definitions

We expressed the WMH volume as a %ICV to standardize to head size. All WMH volumes are %ICV unless stated otherwise. We defined net WMH volume change as the difference between the 1 year and baseline total WMH volumes. We divided the net WMH volume change into quintiles, ranging from quintile 1 (Q1; most WMH volume regression) to quintile 5 (Q5; most WMH volume progression) as previously.^7^ For the sensitivity analysis, we used the percentiles approach^4^ (described in eMethods) as a different definition that groups WMH change into stable, regressing, and progressing WMH.

### Statistical Analysis

We performed all analyses using R version 4.2.2.^28^ with packages *lmer stats* and *FSA*. We tested between-group differences in baseline demographic, clinical, and imaging factors using unadjusted 1-way analysis of variance (ANOVA) with Tukey honestly significance test post hoc tests for continuous variables. For ordinal and nonnormally distributed continuous variables, we applied unadjusted Kruskal-Wallis 1-way ANOVA with post hoc Dunn tests (including Holm adjustment for multiple corrections). For categorical variables, we used unadjusted Pearson χ^2^ tests.

We assessed differences in longitudinal cognitive changes across the quintiles of WMH change over 1 year using repeated measures linear mixed models. In total, we performed 5 linear mixed models, with MoCA total score, TMT-A, TMT-B, TMT B/A ratio, and the LDST as outcomes. The models included a random intercept for the individual participants to allow for interindividual differences. The following predictors, at both time-points, were included age (centered to the mean age at baseline), NIHSS, mRS, PSMD, and quintiles. In addition, TMT-A time was adjusted for in the TMT-B analysis. The models also included baseline only predictors: sex, NART, and WMH volume. After examining assumptions, moderate collinearity between PSMD and baseline WMH volumes were found. The variance inflation factors (VIFs) for PSMD and baseline WMH ranged from 3.10 to 4.82. VIFs for the other variables were all below 2, indicating low collinearity. Repeated analyses without PSMD had the same conclusions. The quintiles of WMH volume change were compared with the reference level of Q3 because Q3 represents the least WMH volume change.

### Sensitivity Analysis

We performed sensitivity analyses on WMH change definition with the 5 linear mixed models by replacing the quintiles with 3 groups defined by the percentile approach in eMethods and eFigure 1. The stable WMH group functioned as a reference group.

### Data Availability

Study protocol is published.^11^ Deidentified participant data can be made available on reasonable email request to the corresponding author.

## Results

**Figure 1:**
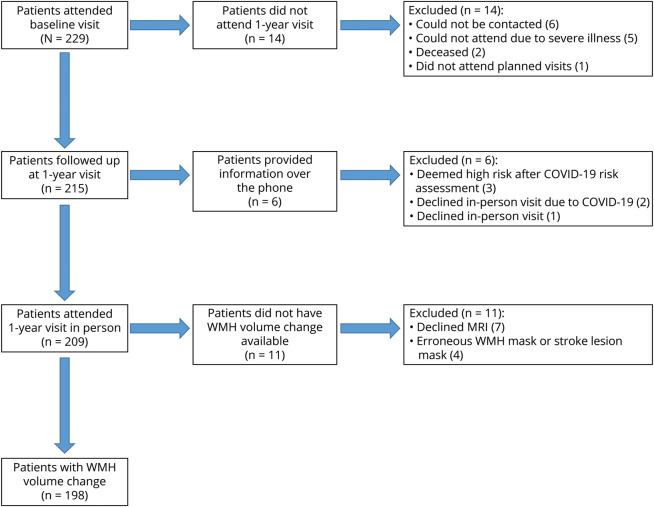
Consort Diagram COVID-19 = coronavirus disease 2019; WMH = white matter hyperintensity.

We included 229 participants; 209 of 229 participants attended both baseline and 1-year visits. WMH volume change data were available for 198 participants (Figure 1, details per outcome eTable 1). At baseline (n = 198), the mean age was 67.54 years (SD = 10.90) and 67% were male. The mean follow-up time was 1.05 years (SD = 0.10). Mean WMH volume change (SD [range]) per quintile was Q1 −1.79 mL (1.54 [−7.98 to −0.68]), Q2 −0.27 mL (0.20 [−0.65 to 0.03]), Q3 0.35 mL (0.18 [0.04–0.70]), Q4 1.43 mL (0.48 [0.75–2.33]), and Q5 5.31 mL (3.07 [2.43–12.84]). Full details of baseline characteristics are in Table 1.

**Table 1:** Baseline Clinical and Imaging Characteristics Per Quintile of WMH Volume Change (%ICV), Q1 Most Regression, Q3 Stable, and Q5 Most Progression of WMH

	n	Q1	n	Q2	n	Q3	n	Q4	n	Q5	*p* Value
Demographics											
Age, y, mean (SD)	40	63.76 (10.13)	40	61.4 (11.4)	40	62.25 (12.06)	39	67.94 (8.95)	39	72.49 (9.25)	<0.001^a^
Sex, male, n (%)	40	30 (75.0)	40	34 (85.0)	40	23 (57.5)	39	23 (59.0)	39	23 (59.0)	0.027^b^
Age leaving fulltime education, mean (SD)	38	16.74 (2.38)	40	17.48 (2.84)	39	18.49 (3.19)	39	16.28 (1.79)	39	17.15 (2.83)	0.010^c^
Stroke subtype, subcortical, n (%)	40	26 (65.0)	40	29 (72.5)	40	15 (37.5)	39	24 (61.5)	39	23 (59.0)	0.023^b^
NIHSS, median (IQR)	40	1 (1–2)	40	1 (0–1)	40	1 (0–2)	39	1 (0–2)	39	2 (1–2)	0.023^c^
mRS, median (IQR)	40	1 (1–1)	40	1 (0–1)	40	1 (1–1)	39	1 (1–1)	39	1 (1–2)	0.642^c^
Hypertension, yes, n (%)	40	32 (80.0)	40	23 (57.5)	40	26 (65.0)	39	28 (71.8)	39	29 (74.4)	0.220^b^
Diabetes mellitus, yes, n (%)	40	5 (12.5)	40	8 (20)	40	5 (12.5)	39	11 (28.2)	39	13 (33.3)	0.083^b^
Hypercholesterolemia, yes, n (%)	40	28 (70)	40	29 (72.5)	40	27 (67.5)	39	25 (64.1)	39	35 (89.7)	0.097^b^
Smoking, current or ex ≤ 1 y, n (%)	40	7 (17.5)	40	7 (17.5)	40	8 (20.0)	39	10 (25.6)	39	5 (12.8)	0.691^b^
MRI measures											
PSMD, mm^2^/s × 10^−3^, mean (SD)	40	0.233 (0.049)	40	0.211 (0.042)	39	0.204 (0.044)	39	0.246 (0.075)	39	0.282 (0.066)	<0.001^c^
WMH volume, mL, mean (SD), range	40	12.68 (12.97) 1.90–70.54	40	8.68 (10.80) 1.05–52.04	40	6.39 (7.68) 0.64–31.65	39	19.51 (23.82) 1.74–97.39	39	30.53 (24.40) 2.58–118.12	<0.001^c^
Cognition											
NART, errors, mean (SD)	40	22.98 (12.87)	40	15.88 (8.14)	39	15.36 (9.19)	38	16.24 (8.02)	39	15.26 (8.45)	<0.001^c^
MoCA, mean (SD)	38	24.76 (3.98)	40	25.43 (2.68)	39	26.31 (2.85)	39	25.77 (3.09)	39	23.38 (4.27)	0.003^a^
TMT-A, s, mean (SD)	39	51.23 (35.12)	40	39.90 (22.12)	40	33.90 (12.26)	39	42.54 (15.93)	39	48.03 (23.73)	0.006^c^
TMT-B, s, mean (SD)	38	130.53 (96.01)	38	99.92 (58.72)	40	90.55 (58.07)	37	114.62 (81.08)	38	158.13 (111.38)	<0.001^c^
TMT B/A ratio, mean (SD)	38	2.65 (0.87)	38	2.66 (1.20)	40	2.63 (1.02)	37	2.83 (1.60)	38	3.26 (1.15)	0.104^a^
LDST, correct, mean (SD)	38	23.13 (7.75)	39	28.03 (7.35)	40	27.15 (7.86)	39	23.10 (7.21)	39	20.97 (7.59)	<0.001^a^

Abbreviations: LDST = Letter Digit Substitution Test; MoCA = Montreal Cognitive Assessment; mRS = modified Rankin score; NART = National Adult Reading Test; NIHSS = NIH Stroke Scale; PSMD = peak width of skeletonized mean diffusivity; TMT = Trail Making Test; WMH = white matter hyperintensity.

All comparisons between quintiles were adjusted for multiple comparisons. Comparisons were not adjusted for covariates. Pairwise comparisons can be found in eTables 2–5.

a One-way analysis of variance.

b χ^2^ test.

c Kruskal-Wallis.

### Demographics, Vascular Risk Factors, and Stroke-Related Measures at Baseline

Participants with more WMH progression (Q4 and Q5) were older (Table 1; additional data are listed in eTables 2–5). Participants in Q5 also had higher NIHSS scores (*H*(4) = 11.34, *p* = 0.023).

There were fewer participants in Q3 with a subcortical stroke subtype (χ^2^(4, n = 198) = 11.36, *p* = 0.023), and those in Q3 left fulltime education at an older age (*H*(4) = 13.27, *p* = 0.010) compared with the other quintiles. Baseline mRS and all vascular risk factors were comparable across quintiles.

### Baseline Imaging Measures

Across the quintiles, baseline WMH volume in milliliters showed a “U-shaped” relation being lowest in Q3, larger in Q1 and Q4, and largest in Q5. PSMD also showed a “U-shaped” relation being lowest in Q3, higher in Q1 and Q4, and highest in Q5 (Table 1, eFigure 2).

### Baseline Cognition

Participants in Q1 made the most errors on the NART, that is, lowest estimate of premorbid intelligence. Across Q1 to Q5, the MoCA showed an “inverse U” pattern, with best scores in Q3 and worse scores in both Q1 and Q5, with Q5 having the worst MoCA scores. A similar pattern was seen for the LDST, most correct in Q3 and fewer correct in Q1 and Q5 (eFigure 3). The TMT-A and TMT-B also show a “U” pattern with fastest times in Q3 and longer times in Q1 and Q5, with Q5 performing worst on TMT-B. However, the “U” pattern for the TMT B/A ratio was flattened for the lower quintiles (eFigure 4).

### Longitudinal Cognitive Changes

#### Change in Global Cognition (Total MoCA Score)

**Figure 2:**
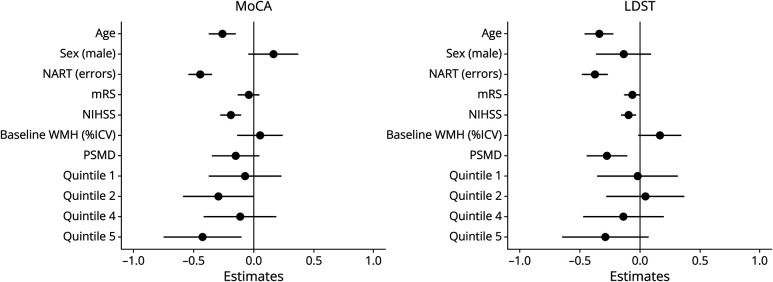
Predictors of Change in MoCA (Left) and LDST (Right) Over 1 Year; Linear Mixed Model Estimates (standardized β) left of line: worsening MoCA or LDST scores; to the right: improving MoCA or LDST scores; higher MoCA and LDST scores reflect better performance. Q3 functions as reference. ICV = intracranial volume; LDST = Letter Digit Substitution Test; mRS = modified Rankin score; NART = National Adult Reading Test at baseline; NIHSS = NIH Stroke Scale; PSMD = peak width of skeletonized mean diffusivity; Q = quintile of WMH change; Q1 = most WMH regression; Q5 = most WMH progression; WMH = white matter hyperintensity.

Worsening MoCA scores over 1 year were associated with increasing age (standardized β [95% CI] −0.256 [−0.367 to −0.144]), more errors on the baseline NART (β −0.455 [−0.552 to −0.357]), larger increase in WMH %ICV (Q5 compared with Q3; β −0.428 [−0.750 to −0.106]), worsening NIHSS (β −0.188 [−0.275 to −0.101]), but not baseline WMH volume or PSMD (Figure 2, eTable 6).

#### Change in Processing Speed (LDST)

Decreasing LDST scores were associated with increasing age (β −0.339 [−0.458 to −0.220]), more baseline NART errors (β −0.374 [−0.482 to −0.267]), worsening NIHSS (β −0.094 [−0.155 to −0.033]), and increasing PSMD (β −0.274 [−0.443 to −0.104]) (Figure 2, eTable 6). There was a suggestive trend across the quintiles with Q5 showing the greatest decline in LDST performance.

#### Change in Processing Speed and Executive Function (TMT-A, TMT-B, and TMT B/A Ratio)

**Figure 3:**
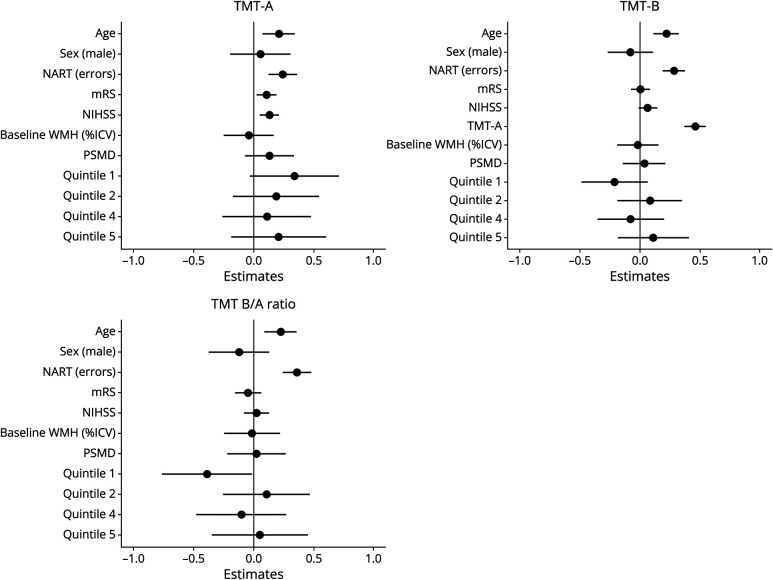
Predictors of Change in TMT-A (Top Left), TMT-B (Top Right), and TMT B/A Ratio (Bottom Left) Over 1 Year; Linear Mixed Model Estimates (standardized β) left of line: decreasing times or B/A ratio; to the right: increasing times or B/A ratio. Longer times or higher B/A ratio indicates poorer performance. Q3 functions as reference. ICV = intracranial volume; mRS = modified Rankin score; NART = National Adult Reading Test at baseline; NIHSS = NIH Stroke Scale; PSMD = peak width of skeletonized mean diffusivity; Q = quintile of WMH change; Q1 = most WMH regression; Q5 = most WMH progression; TMT = Trail Making Test; WMH = white matter hyperintensity.

Over 1 year, increasing time on the TMT-A was associated with increasing age (β 0.213 [0.080–0.347]), more errors on the baseline NART (β 0.247 [0.127–0.366]), worsening mRS (β 0.110 [0.027–0.194]), and worsening NIHSS (β 0.135 [0.056–0.214]) (Figure 3, top left) but not baseline WMH volume or PSMD. There was no difference between WMH change quintiles (eTable 6).

Increasing time on the TMT-B was associated with increasing age (β 0.221 [0.117–0.325]), more errors on baseline NART (β 0.285 [0.192–0.375]) and increasing times on the TMT-A (β 0.466 [0.378–0.554]) but not baseline WMH volume or PSMD (Figure 3, top right).

Increasing TMT B/A ratio, that is, worsening executive performance, was associated with increasing age (β 0.227 [0.089–0.365]), and more errors on the baseline NART (β 0.359 [0.239–0.479]). Decreasing TMT B/A ratio was associated with WMH volume regression (Q1 compared with Q3; β −0.385 [−0.758 to −0.012]) (Figure 3, bottom left). The suggestive trend seen across quintiles in TMT-B (Figure 3, top right) becomes more apparent when executive function is corrected for processing speed using the TMT B/A ratio.

### Sensitivity Analysis

We repeated the analyses using WMH change defined by percentile (eMethods and eTable 7). The overall pattern was similar for longitudinal MoCA change regarding age, NART, and NIHSS. There was no clear association with WMH progression. The TMT-B results were similar for the quintile and percentile definitions. WMH regression was associated with increasing TMT-A (β 0.338 [0.005–0.672]), and a lower TMT B/A ratio over time compared with stable WMH (β −0.327 [−0.668 to −0.015]), similar to the association found with quintiles (β −0.385 [−0.758 to −0.012]). The percentile approach was also consistent for the LDST where participants with more WMH progression showed worsening performance compared with those with stable WMH (β −0.370 [−0.652 to −0.089].

## Discussion

We compared baseline clinical and imaging characteristics and differences in longitudinal cognitive outcomes across longitudinal WMH change trajectories. We show that participants with the most WMH progression and regression had large WMH volumes and poorer cognitive performances at baseline, seen in the “U-shaped” patterns for baseline WMH volume, TMT-A and TMT-B, and inverse “U-shaped” pattern for MoCA and LDST (eFigures 2–4). Participants with the most WMH progression (Q5) showed global cognitive decline (MoCA), while participants with most WMH regression showed improvement in executive function and retained global cognitive function at 1 year. These findings were robust to a different WMH change definition and add cognitive relevance to MRI markers of improved tissue health that we demonstrated previously in the same population.^10^

Characteristics of participants with most WMH progression, that is, older age,^5^ larger baseline WMH volume,^3,8^ worse baseline cognitive function,^2,29^ and worse baseline microstructural integrity (PSMD),^8,30^ are in line with previous studies. Our analysis confirmed a younger age at time of stroke for participants with most WMH regression.^8^ However, they were also younger when leaving fulltime education and had lower premorbid intelligence. This has not been apparent in previous WMH change studies^4,8^ but reflects our study population of minor strokes because lower childhood/premorbid IQ and education associate with increased risk of stroke including at younger ages.^31^

Participants with WMH regression may have better executive functioning over time, compared with people with stable WMH. This was mainly based on the TMT B/A ratio and confirmed by the sensitivity analysis. The B/A ratio was proposed as a sensitive measure of executive functioning because it is corrected for processing speed.^17^ A previous study did not find a difference in executive function between people with WMH regression and stable WMH, possibly due to using a composite score.^32^ We did not find a difference between WMH regression and stable WMH for global cognition because global cognition remained relatively stable in both. However, participants with WMH regression should have had worse (i.e., deteriorated) global cognition at 1 year based on their high baseline WMH volume, suggesting that the WMH regression interrupted the expected decline in global cognition.^1^ Different results and directions of results between WMH regression and WMH progression suggest that WMH regression can occur^3,8^ and can have important clinical implications.

It is interesting that the LDST is the only outcome associated with PSMD, indicating that deteriorating white matter structure is related to worse processing speed, confirmed by the sensitivity analysis. Previous studies show that the LDST, or other processing speed tests including the TMT-A, is associated with higher PSMD in older people, cerebral amyloid angiopathy, Alzheimer disease, and genetic and sporadic SVD,^33^ but this was based on a compound score for cognitive domains that included the LDST and often also included the TMT-A and TMT-B.^33^ We did not find clear associations between PSMD and either TMT-A, TMT-B, or B/A ratio.

Strengths of this study include the large sample, high retention, testing several aspects of cognitive function, applying different WMH change definitions, and performing a sensitivity analysis to validate results. Our longitudinal analyses were corrected for age, sex, and premorbid intelligence because these variables influence cognitive outcomes,^34^ which was not always performed in other studies.^4,9,35^

Limitations of the study are the use of a single cognitive screening test, with 2 additional tests, rather than using complete cognitive batteries to assess all relevant cognitive domains at both visits. Although all participants experienced a nondisabling stroke and did not require inpatient rehabilitation, some participants might have experienced residual hand-weakness of their dominant hand that might have slightly influenced their performance on the TMT and LDST, with very minimal effect on the MoCA and TMT B/A ratio,^17^ and the results need to be interpreted with caution. In our longitudinal analyses, we compared quintiles of regression and progression with the quintile of most stable WMH. We did not directly compare the quintile with most regression to the quintile with most progression. This might affect interpretation and generalizability of the findings. However, we did perform sensitivity analyses that confirmed our findings. Longer follow-up times, including multiple visits, need to be explored as WMH volumes change in a nonlinear fashion,^3^ and long follow-up times might affect the interpretation of what we currently consider to be “stable” WMH. Findings are limited to minor ischemic stroke patients, and further examination in other populations, for example, severe strokes, memory clinics, and “healthy” people is required. We did not assess influence of stroke subtype, for example, Q3 had a higher proportion of cortical strokes, which might reflect the stronger association of WMH with lacunar stroke; a stroke subtype analysis might be of interest in future research. Future studies should also use neuropsychological tests, and potentially apply composite scores, to assess all cognitive domains to strengthen interpretations of the outcomes. There is a pressing need for consensus on WMH change definitions to facilitate comparability of results across studies. This would provide a more stable measure for clinical trials and help to rationalize data on so-called “normal” WMH volumes by age. In addition, it would be interesting to assess whether WMH change definitions can be applied to different populations and if they relate to similar cognitive outcomes. Any influence of prescribed medications would be relevant to examine because this might affect clinical practice. Previously, we analyzed tissue signatures of WMH change using QT1, fractional anisotropy, mean diffusivity and neurite orientation dispersion, and density imaging measures,^10^ but future studies should assess a wider range of in vivo imaging tissue signatures to identify the underlying processes of changing WMH in more detail.

People with the most WMH progression and regression have the largest WMH volume changes and worst microstructural integrity at baseline, suggesting more brain vascular “instability.” However, there are different cognitive trajectories because the largest WMH progression seems to lead to overall worse cognitive outcomes, while, despite having a high baseline WMH volume, WMH regression is associated with improving executive functioning and similar global cognitive trajectories to people with more stable WMH. The signs that WMH regression might benefit executive functioning and stabilize global cognitive decline are encouraging and provide a potential but important intervention target.

## Glossary

ANOVA: analysis of variance
FLAIR: fluid-attenuated inversion recovery
ICV: intracranial volume
LDST: Letter Digit Substitution Test
MoCA: Montreal Cognitive Assessment
NART: National Adult Reading Test
NIHSS: NIH Stroke Scale
PSMD: peak width of skeletonized mean diffusivity
SVD: small vessel disease
TMT: Trail Making Test
VIF: variance inflation factor
WMH: white matter hyperintensity
